# Molecular genotyping reveals mixed bovine and human trypanosomiasis in cattle from West Africa

**DOI:** 10.14202/vetworld.2023.149-153

**Published:** 2023-01-23

**Authors:** Olanrewaju B. Morenikeji, Jessica L. Metelski, Anastasia Grytsay, Jacob Soulas, Mabel O. Akinyemi, Bolaji N. Thomas

**Affiliations:** 1 Division of Biological Health Sciences, University of Pittsburgh, Bradford, PA, 16701, United States; 2 Department of Biomedical Sciences, Rochester Institute of Technology, Rochester, NY 14623, United States; 3 Department of Biological Sciences, Fairleigh Dickinson University, Madison, NJ 07940, United States

**Keywords:** Africa, animal, genotyping, internal transcribed spacer, trypanosome

## Abstract

**Background and Aim:**

Animal trypanosomiasis is a major contributor to agricultural and economic losses, especially in sub-Saharan Africa. We have shown that some animal species expressed genes that are significant players in immune response to bovine trypanosomosis, impeding signs and symptoms of the disease. We hypothesize that such animals are contributors to disease transmission dynamics and severe outcomes. Therefore, this study aims to ascertain trypanosome species diversity in cattle and their potential role as reservoirs for the transmission of human disease.

**Materials and Methods:**

We performed a molecular genotyping of trypanosome internal transcribed spacer 1 (ITS-1) and 18S ribosomal RNA genes on genomic DNA extracts from randomly sampled N’Dama cattle from slaughterhouses in Nigeria. We identified trypanosome species circulating among the animals through polymerase chain reaction and genomic sequencing. We performed multiple sequence alignments as well as conducted a phylogenetic relationship between identified species.

**Results:**

In all, 9 of 127 (7.1%) samples were positively amplified (band sizes ranging from 250 bp to 710 bp), including an isolate with two distinct bands (700 and 710 bp), indicating two trypanosome types. Sequence similarity and homology analysis identified four species, namely: *Trypanosoma* *vivax*, *Trypanosoma congolense* forest type, *T. congolense* savannah type, and *Trypanosoma brucei*. Interestingly, one of the bands, additionally verified by nucleotide sequencing, was identified as a human trypanosome (*Trypanosoma brucei gambiense*), confirming our hypothesis that cattle are potential reservoir hosts for human trypanosomes.

**Conclusion:**

Overall, we observed different trypanosome species in our study area, with animals on the same farm infected with multiple species, which could complicate treatment and disease control strategies. Finding human trypanosome species strengthens the argument that disease transmission dynamics are modulated by other vertebrates, further complicating control programs.

## Introduction

Nagana is a fatal cattle disease in sub-Saharan Africa caused by different species of trypanosome (Protozoa: Sarcomastigophora). Although there are different species of trypanosomes, not all of them are pathogenic in mammalian species. Five species of trypanosomes are pathogenic in cattle: *Trypanosoma* *congolense*, *T. congolense* savannah type, *T. congolense* forest type, *Trypanosoma vivax*, and *Trypanosoma brucei* [1, 2]. Others may use cattle as reservoirs of infection, but are non-pathogenic in cattle. On the other hand, sleeping sickness, also referred to as human African trypanosomiasis, is a blood-borne infection caused by *Trypanosoma brucei* *gambiense* or *Trypanosoma brucei rhodesiense*. The infection is transmitted by reservoir hosts, such as cattle to humans through the bite of tsetse flies (*Glossina* spp.) [3]. The previous studies [4–7] have shown that certain cattle breeds, such as N’Dama are trypanotolerant with the ability to control parasitemia and anemia, thus harboring the parasite and serving as reservoir hosts. This is thought to be an evolutionary adaptation that the N’Dama breed acquired, upon exposure to trypanosomiasis after being introduced to sub-Saharan Africa.

When the tsetse fly consumes a blood meal from a trypanosome-infected animal, the parasites having undergone developmental changes within the fly, are deposited into the bloodstream of the new host, such as a human, during another blood meal [8]. These asymptomatic disease-tolerant animals (N’Dama), we hypothesize, could serve as reservoirs for zoonotic disease transmission, implying a need to screen such for pathogen and species diversity. We hypothesize that such cattle are significant contributors to bovine disease transmission dynamics in sub-Saharan Africa and are potential reservoir hosts for human trypanosomes, adding to the challenges of disease control and drug resistance. Hence, there is a need to identify these human parasites within the cattle population, potentially delineating the inter-species dynamics between pastoralists and animals.

This study aimed to perform the molecular characterization of animal and human trypanosomes using the internal transcribed spacer 1 (ITS-1) and 18S ribosomal RNA genes in 127 genomic DNA extracts from randomly sampled cattle in Nigeria and elucidate their phylogenetic relationship.

## Materials and Methods

### Ethical approval

Ethical approval is not applicable for this study. Our samples were from discarded blood from slaughterhouses.

### Study period and location

The study was conducted from 03-05-2018 to 17-07-2018 at a slaughterhouse in southwestern Nigeria.

### Sample collection

Blood samples (10 mL each) were collected during slaughter from 127 apparently healthy N’Dama cattle. Genomic DNA was extracted from the blood samples with the QIAamp DNA Mini Kit (Qiagen, Carlsbad CA), following the manufacturer’s protocol.

### Molecular identification

Two sets of specific 18S ribosomal RNA gene primer pairs were used for a polymerase chain reaction (PCR) to amplify various species of trypanosomes, including human trypanosomes (*T. brucei gambiense* or *T. brucei rhodesiense*), as previously described [9, 10]. The primer pairs detail is shown in Table-1.

**Table-1:** Primer pairs for PCR amplification and DNA sequencing.

S. No.	Gene name	Primer name	Primer sequence (5’- 3’)
1	Internal transcribed spacer 1 (ITS1) RNA gene primer pairs [10]	AITS Forward	CGGAAGTTCACCGATATTGC
		AITS Reverse	AGGAAGCCAAGTCATCCATC
2	18S ribosomal RNA gene primer pairs [9]	M18S-II-F-Tb (*T. brucei gambiense*)	CGTAGTTGAACTGTGGGCCACGT
		M18S-II-R-Tb (*T. brucei gambiense*)	ATGCATGACATGCGTGAAAGTGAG

PCR=Polymerase chain reaction, *T. brucei=Trypanosoma brucei*

A PCR reaction mixture composed of 1 μL of both forward and reverse ITS-1 primer, 12.5 μL of Lucigen EconoTaq PLUS Green 2X Master Mix, 9.5 μL of PCR-grade water and 1 μL of template DNA, making a total reaction volume of 25 μL. The reaction mixture was set up in 96 well PCR plates and programmed as follows: 95°C for 10 min and 35 cycles 95°C for 50 s, 60°C for 30 s, 72°C for 50 s, and the final extension 72°C for 5 min. Post-amplification, 5 μL of the PCR product was examined on 2% ethidium bromide-stained agarose gel and viewed with a BioDoc-It Imaging System; band sizes were determined with a 100 base pair DNA ladder, as described by Thomas *et al*. [11]. Trypanosome positive PCR products were purified for sequencing using the QIAquick PCR Purification Kit, following the manufacturer’s protocol (Qiagen Inc., Valencia, CA, USA).

### Sequence processing and analysis

Bidirectional sequencing of purified PCR products was performed at a commercial facility (Genewiz, South Plainfield, NJ, USA). Sequences were processed for quality control, and contigs were assembled with Lasergene, version 4.0 (DNAStar, Madison, WI, USA). To further confirm our identified trypanosomes, we queried the GenBank database (https://www.ncbi.nlm.nih.gov/genbank/) with our sequences for similarity and retrieved the homologous sequences. Our sequences were deposited in GenBank, and accession numbers (MN213746 - MN871764) were assigned. We performed a multiple sequence alignments with MEGA and constructed a phylogenetic tree with the unweighted pair group method with Unweighted Pair Group Method with Arithmetic Mean (UPGMA) [12, 13].

## Results

**Figure-1:**
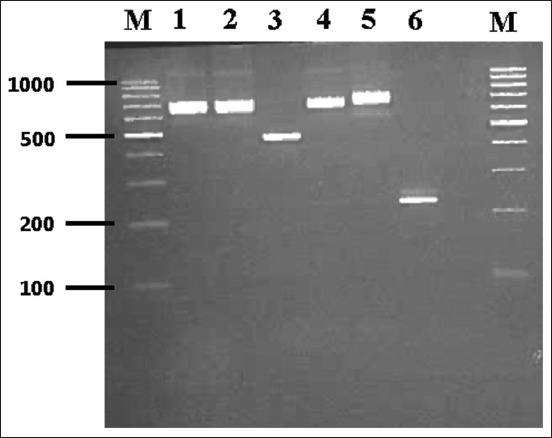
Gel electrophoresis of polymerase chain reaction products showing genomic DNA amplified for the internal transcribed spacer-1 gene. Agarose gels stained with ethidium bromide depicting various and multiple animal trypanosome species and mixed human trypanosomes. M: 100 base pair DNA ladder, where the 500 bp band stains most intensely (Thermo Fisher Scientific); lanes 1, 2, and 4: 700 bp (representing *Trypanosoma congolense*); lane 3: 520 bp (representing *Trypanosoma brucei gambiense*); lane 5: 710 bp (representing *T. congolense* forest); lane 6: 250 bp (representing *Trypanosoma vivax*).

**Figure-2:**
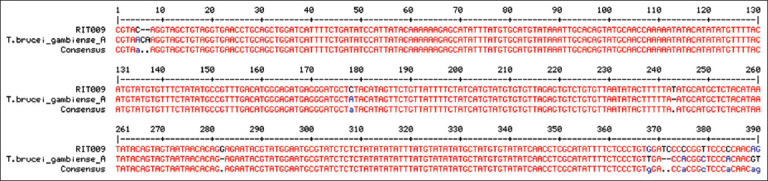
Pairwise nucleotide sequence alignment of our RIT009 isolate with *Trypanosoma* *brucei gambiense* (accession number AF306774.1), a human isolate from Cote D’Ivoire, confirming the taxonomy of our isolate.

**Figure-3:**
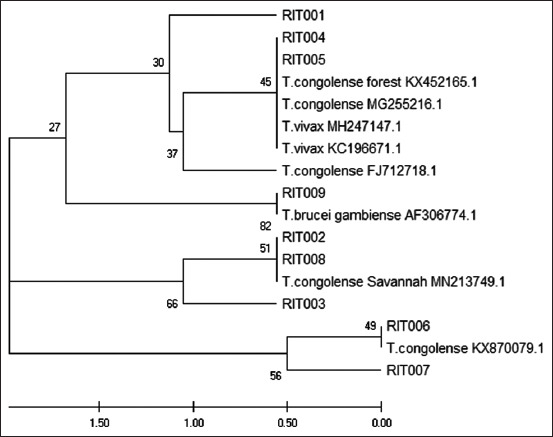
Evolutionary relationships of taxa. The evolutionary history was inferred using the UPGMA method [12, 13]. The optimal tree with the sum of branch length = 18.70498668 is shown. The tree is drawn to scale, with branch lengths in the same units as those of the evolutionary distances used to infer the phylogenetic tree. The evolutionary distances were computed using the number of differences method and are in the units of the number of base differences per sequence. The analysis involved 15 nucleotide sequences. All positions containing gaps and missing data were eliminated. There were a total of 46 positions in the final.

From our PCR results, 9 (7.1%) of 127 bovine samples were positively amplified for trypanosomes, with band sizes ranging from 250 bp to 710 bp (Figure-1). Interestingly, our analysis showed an animal with multiple trypanosome species (*T. congolense* savannah type and *T. congolense* forest type) and another animal possibly infected with a human trypanosome (*T. brucei*) (Figure-1). Through sequence analysis and NCBI BLAST search, we confirmed the similarity of our sequence with those in GenBank, validating our PCR results. In addition to the species already identified through PCR and gel electrophoresis analysis, multiple sequence alignment and phylogenetic analyses show *T. brucei gambiense* to be closely related to one of our isolates (RIT009) (Figure-2). Furthermore, phylogenetic analyses revealed extensive disparity in the clustering of trypanosome species in our study location (Figure-3) [12, 13]. We found that isolates RIT001, RIT004, and RIT005 clustered with *T. vivax* and *T*. *congolense forest* type, isolates RIT002, RIT003, and RIT008 were close to *T. congolense* savannah type, and isolates RIT006 and RIT007 were most closely related to *T. congolense*. Significantly, and potentially of clinical relevance, isolate RIT009 is closely related to *T. brucei gambiense*, the human species, as shown on the phylogenetic tree (Figure-3).

## Discussion

The low prevalence of trypanosomosis in this study is surprising when placed in context with other published reports [10, 14, 15]. This possibly attests to the effectiveness of current control strategies, the efficiency of veterinary inspection officers who remove infected animals from the pool during the slaughter process, or incomplete data from previous studies producing high prevalence rates. It is also possible that our limited sample size or the genotyping method, known to produce inconsistent results [16], may have contributed to the low prevalence rates. Based on the previous reports [17–20] and the expected band sizes, we identified *T. vivax*, *T. congolense* forest type, *T. congolense* savannah type, and *T. brucei*. The demonstration that *T. congolense* is the predominant species in our study area is unexpected. This is at variance with previous reports on the prevalence of *T. vivax* in several cattle species in endemic areas. The present observation could be attributed to the shorter lifecycle of the species in tsetse fly proboscis and rapid multiplication in the host [17–20]. *T. congolense* has also been reported in some human infections with animal trypanosomes [21]. Our results are corroborated by other studies [22–24], also implying the need for further species deconvolution. The observation of mixed *T. congolense* savannah and forest types may be of future clinical significance among these animals, thus implying a need for constant epidemiologic surveillance.

Such mixed animal infections are becoming common occurrences globally, especially in areas with significant endemicity for bovine trypanosomosis, as recorded in East Africa [25, 26]. However, it is somewhat surprising that we reported this finding in cattle, since previously reported multiple infections have been in goats or pigs [18]. Such multiple trypanosome species in animals complicate treatment and disease control plans in endemic areas and an additional hurdle. This observation calls for further animal testing, advisedly with very sensitive molecular genotyping tools, in these environments.

Few reports of mixed infection with human trypanosomes have been recorded, making this observation concerning, especially considering the close living quarters between cattle and herders/pastoralists [25, 26]. This suggests that these animals may serve as reservoir hosts for human infections and may be involved in the cycle of human infection with trypanosomiasis. Our isolate matched perfectly with Dal972 (accession number: AF306774.1), a human *gambiense* isolate from Cote D’Ivoire [27], further confirming our observation of mixed human and animal trypanosomes.

Since the tsetse flies that transmit the infection are very close to the animals, and the pastoralists live in close quarters with these animals, we can surmise that these pastoralists are probably infected, yet show no signs of infection. The possibility of asymptomatic subclinical infection among these pastoralists or their serving as reservoir hosts contributing to the extensive distribution of trypanosomes deserves further immunogenetic elucidation, considering interethnic observation between Mossi and Fulani tribes, when exposed to malaria infection [28]. Dissecting this observation between pastoralist population groups of East and West Africa could potentially shed light on current trypanosome control strategies and would be of urgent importance.

## Conclusion

Our molecular genotyping identified trypanosome species diversity and multiple mixed infections in cattle, revealing the possible cause of diverse clinical signs of disease, making treatment difficult and complete disintegration of instituted national control programs. Importantly, we found an isolate RIT009 closely related to the human isolate *T. brucei gambiense*, thus confirming that trypanotolerant N’Dama could serve as a reservoir host and aid human trypanosome transmission. Although the sample size in this study is relatively small and may have contributed to a few detections of human trypanosome isolate as seen, a similar study with a larger sample size is recommended. In the future, we will examine circulating trypanosome species among pastoralists and other local mammals to clarify the population genetics of the parasite and potential interspecies transmission or protection, as well as decipher the possible role of seasonality on circulating trypanosome species.

## Authors’ Contributions

OBM and BNT: Conceptualized the research project, designed the project, and developed the methodology for the study. JLM, OBM, and BNT: Administered the project and oversaw the writing of the original draft. JLM, AG, JS, MOA, BNT, and OBM: Contributed to critically reviewing the paper and developing the final draft. All authors have read and approved the final manuscript.
